# Supplementary material for: Reproductive Outbreaks of Sogatella furcifera Mediated by Overexpression of the Nuclear Receptor USP under Pressure from Triflumezopyrim
Source: Int J Mol Sci. 2022 Nov 9;23(22):13769. doi: 10.3390/ijms232213769 (PMC9698614; doi:10.3390/ijms232213769)
Supplement: Supplementary file 1 [file ijms-23-13769-s001.zip › ijms-1938694-supplementary.pdf]

Table S1. The Primer used in the research

Purpose	Primer name	Primer sequence(5’ →r’）	Tm	Product length
**qPCR**	RPL9-F	TGTGTGACCACCGAGAACAACTCA	65.0℃	142
	RPL9-R	ACGATGAGCTCGTCCTTCTGCTTT	65.8℃	
	qUSP-F	CATCACTCATGGGGACGAGG	60.6℃	176
	qUSP-R	GTCCGGCTTCATTTCCAACG	62.7℃	
	qKr-h1-F	GTCGCCTGTGCCTTCTAGTT	57.0℃	165
	qKr-h1-R	AGCGGATGCACCTGATACTG	58.3℃	
	qEcR-F	GCCCAGTCAGCTCATCCAAT	59.7℃	118
	qEcR-R	CTGGGGTAGCTCGGGATTTC	61.0℃	
	qPOU-F	GACAGTGGCACAATCCAAGC	58.5℃	139
	qPOU-R	GCCCTGTGAATAGGTCACCC	59.0℃	
	qPDZ-F	TGGCGTTGTTCTACACGGAA	59.8℃	117
	qPDZ-R	GCGAGATCTTCCAAGCCAGT	59.2℃	
	qDpp-F	AGCTCCGGTGGAAACAATCT	58.6℃	163
	qDpp-R	TGGCGCCTTTCTGCTTTTTC	62.9℃	
	qTbox6-F	ATACATCCACCCCGATTCGC	61.9℃	90
	qTbox6-R	AGCGTGTGATTGGTGAGCTT	57.9℃	
	qVg-F	TTATCGCAGTTGCCGGAGTT	60.8℃	129
	qVg-R	TACCCACCATCTGGGGTGAT	59.5℃	
	qVgR-F	ACTTTACCGGATGGCGGATG	62.2℃	148
	qVgR-R	CCGGCTCCAGACAGAAGTTT	59.2℃	
	qChi8-F	GGCCTTGACTTGGATTGGGA	62.0℃	134
	qChi8-R	CCTATGGCAGCCGTCAGAAT	60.1℃	
	qGPCR A22-F	GACTGGACAGCTCTCGCTTT	56.9℃	175
	qGPCR A22-R	CCGCATCCGCTGTAGATGAA	61.8℃	
	qGPCR A39-F	CATATCGGTGTCAGCCGGAA	61.5℃	118
	qGPCR A39-R	GATGACGGAGGCCACACTAC	57.4℃	
**PCR**	SfUSP-F	AGAAACCGATGATGTCTGTGAC	56.4℃	1263
	SfUSP-R	TTAGGGATCTGTGGGCGCTT	62.0℃	

	SfKr-h1-F	TCACCAGACACTATCGCACG	57.4℃	1115
	SfKr-h1-R	CAGCGGATGCACCTGATACT	58.3℃	
**RNAi**	dsUSP-F	TAATACGACTCACTATAGGCCAGTG GCAAGCACTATGGA	77.8℃	500
	dsUSP-R	TAATACGACTCACTATAGGAACTGA CCGGTGCGAGAAC	76.5℃	
	dsKr-h1-F	TAATACGACTCACTATAGGTCAAAGT GCACAGTCGGACG	77.0℃	500
	dsKr-h1-R	TAATACGACTCACTATAGGCTGCGGT GAGCGAGGATAC	77.7℃	
	dsGFP-F	TAATACGACTCACTATAGGGAAGGG CGAGGAGCTGTTCACCG	82.7℃	700
	dsGFP-R	TAATACGACTCACTATAGGGCAGCA GGACCATGTGATCGCGC	84.4℃	

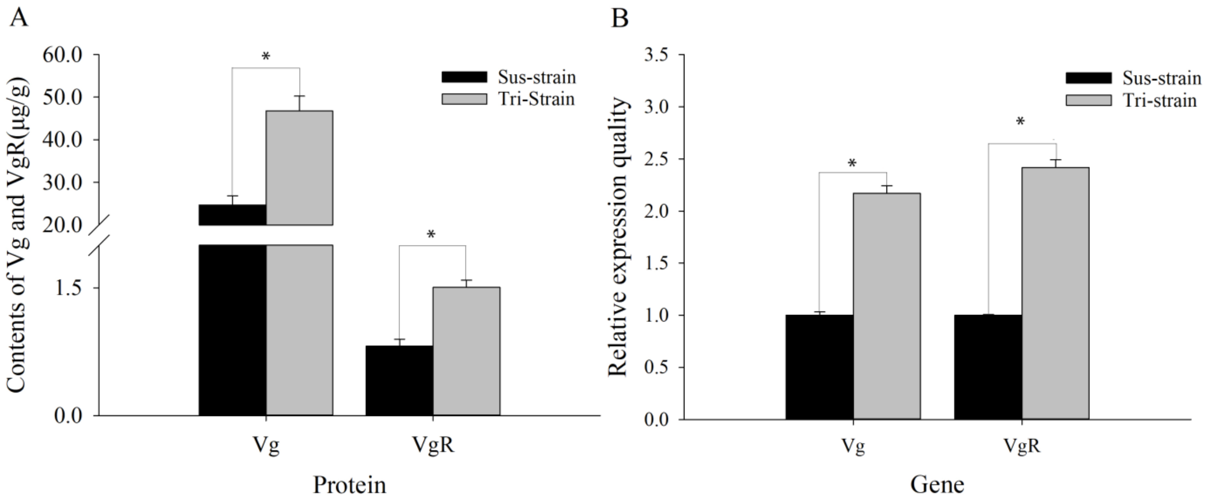

Figure S1 Content of *Vg* and *VgR*, A, The protein contents of Vg and VgR in the female adults of Sus- and Tri-strain. B, The relative expression qualities of *Vg* and *VgR* genes in the female adults of Sus- and Tri-strain. * showed significance at 0.05 levels with tukey's post-hoc tests, respectively.

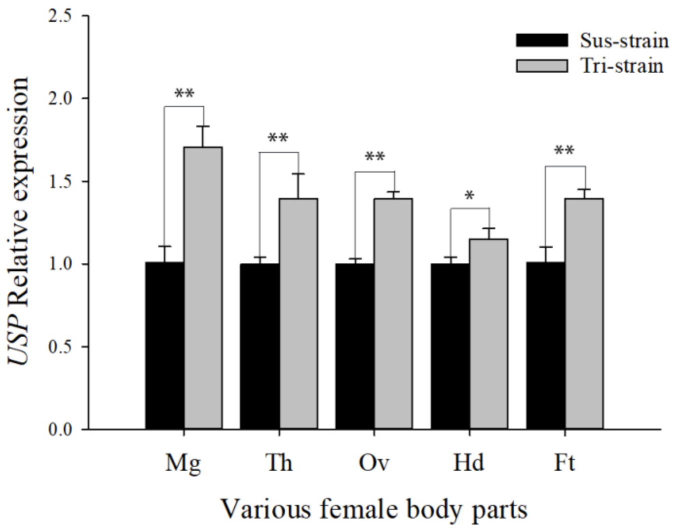

Figure S2 The relative expression levels of USP in Tri-strain various parts of the female adult compared with Sus-strain. Mg, midgut. Th, thorax. Ov, ovarian. Hd, head. Ft, feet. * and ** showed significance at 0.05 and 0.01 levels with tukey's post-hoc tests, respectively.

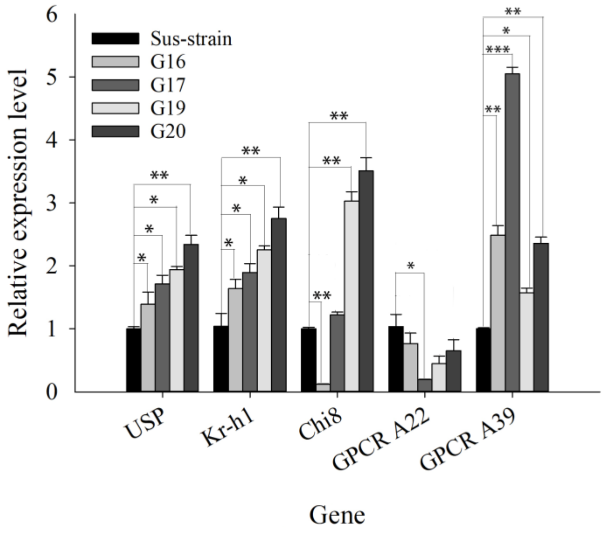

Figure S3 Relative expression levels of growth and reproduction-related genes in the third instar nymph. *, ** and *** showed significance at 0.05, 0.01 and 0.001 levels with tukey's post-hoc tests, respectively.

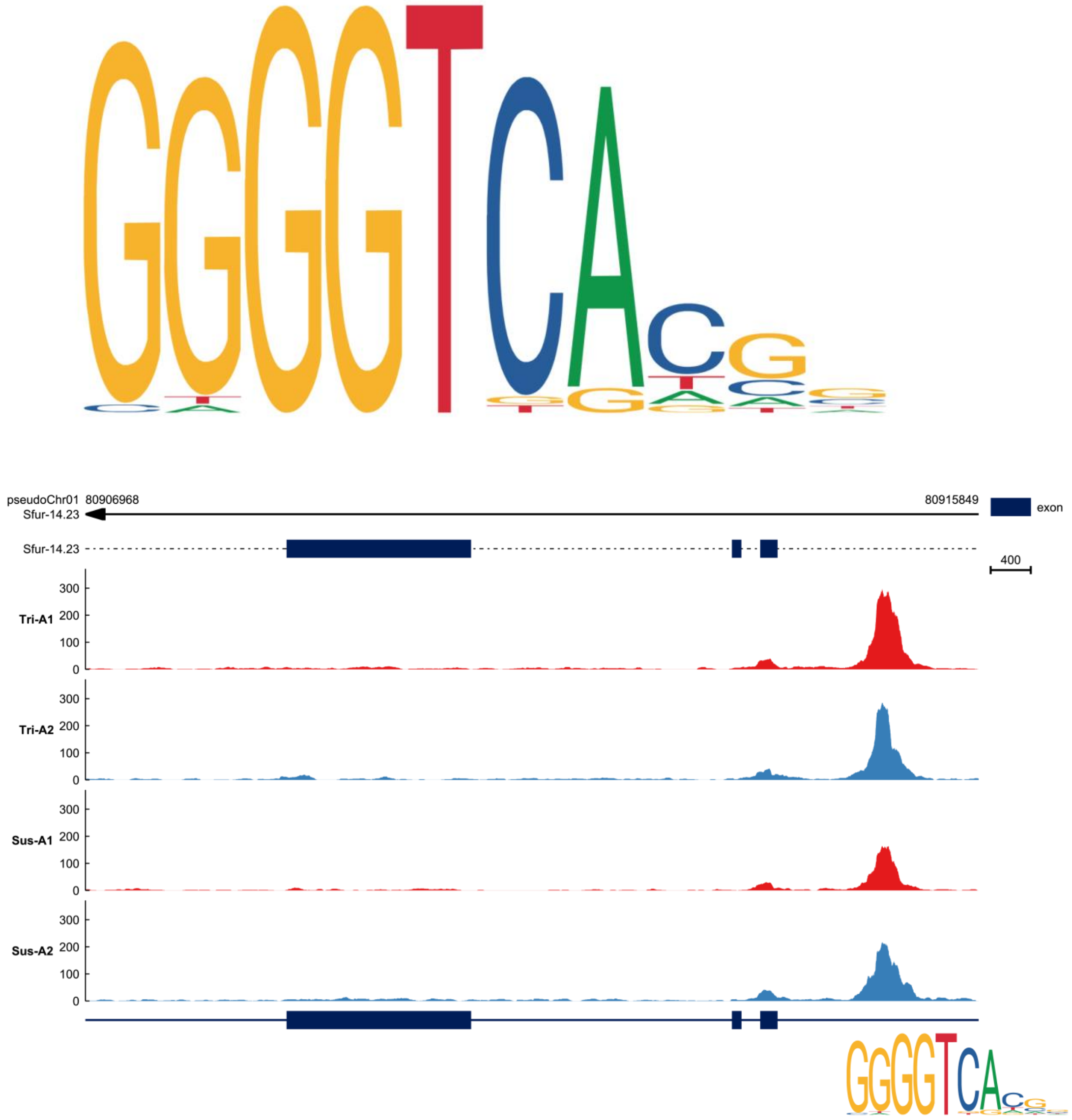

Figure S4 Binding sequence prediction of transcription factor USP association regulation related genes Kr-h1 of S. furcifera.

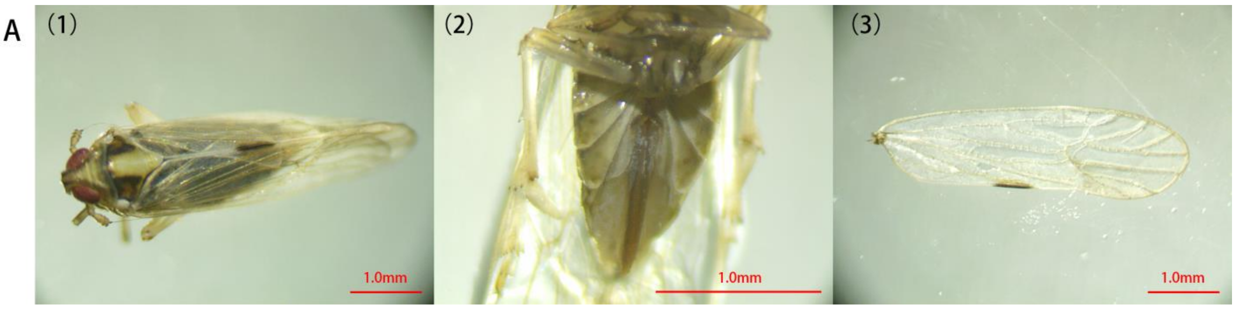

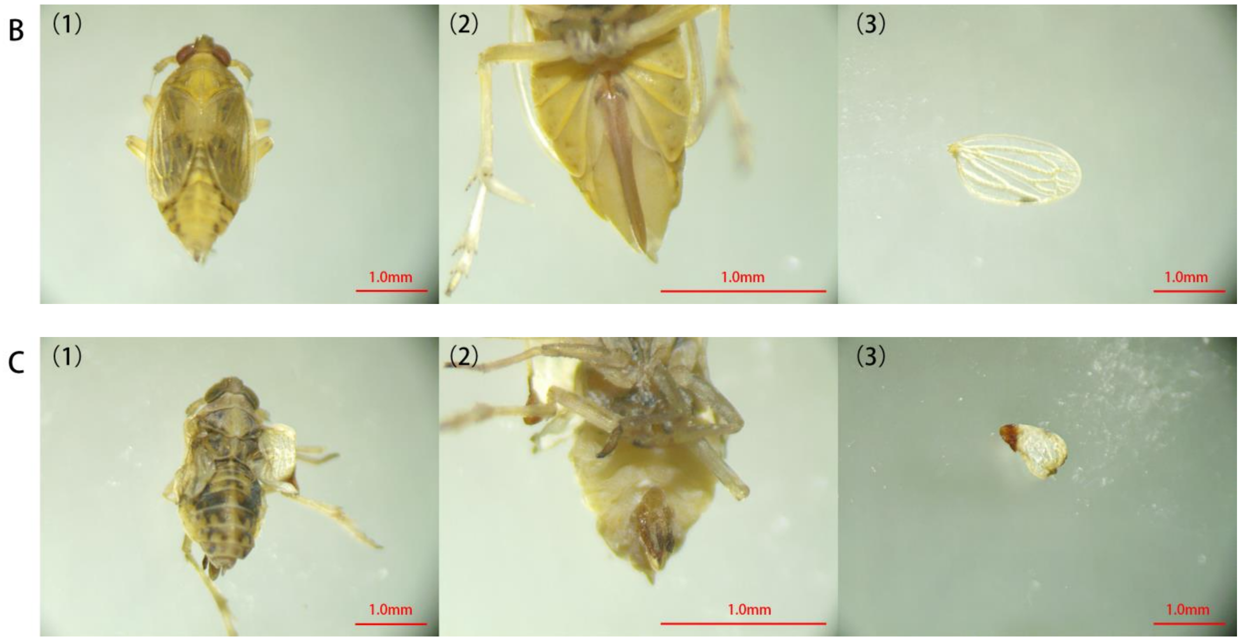

Figure S5 The phenotypes of female adults after *Kr-h1* RNAi. A, phenotypes of long-winged female adults treated with dsGFP. B, phenotypes of short-winged female adult treated with ds*GFP*. C, phenotypes of female adults treated with ds*Kr-h1*. (1), the appearance of the whole adult. (2), the phenotype of the female adult genitalia. (3), the wing type of the female adult. Scale bar, 1.0mm.
